# Selective SERCA2a activator as a candidate for chronic heart failure therapy

**DOI:** 10.1186/s12967-024-04874-9

**Published:** 2024-01-19

**Authors:** Martina Arici, Shih-Che Hsu, Mara Ferrandi, Paolo Barassi, Carlotta Ronchi, Eleonora Torre, Andrea Luraghi, Gwo-Jyh Chang, Patrizia Ferrari, Giuseppe Bianchi, Francesco Peri, Antonio Zaza, Marcella Rocchetti

**Affiliations:** 1 Department of Biotechnology and Biosciences, Università Degli Studi di Milano-Bicocca, P.Za Della Scienza 2, 20126 Milan, Italy; 2 CVie Therapeutics Limited, Taipei, 11047 Taiwan; 3 Windtree Therapeutics Inc, Warrington, PA 18976 USA; 4 Chang Gung University, Tao-Yuan, 333323 Taiwan; 5 Università Vita-Salute San Raffaele, 20132 Milan, Italy

**Keywords:** SERCA2a, Istaroxime, PST3093, Heart failure, Diastolic dysfunction, STZ

## Abstract

**Background:**

The sarcoplasmic reticulum (SR) Ca^2+^ ATPase (SERCA2a) depression substantially contributes to diastolic dysfunction in heart failure (HF), suggesting that SERCA2a stimulation may be a mechanism-based HF therapy. Istaroxime is a drug endowed with both a SERCA2a stimulatory activity and a Na^+^/K^+^ pump inhibitory activity for acute HF treatment. Its main metabolite PST3093 shows a more favorable therapeutic profile as compared to the parent drug, but it is still unsuitable for chronic usage. Novel PST3093 derivatives have been recently developed for oral (chronic) HF treatment; compound 8 was selected among them and here characterized.

**Methods:**

Effects of compound 8 were evaluated in a context of SERCA2a depression, by using streptozotocin-treated rats, a well-known model of diastolic dysfunction. The impact of SERCA2a stimulation by compound 8 was assessed at the cellular level ad in vivo, following i.v. infusion (acute effects) or oral administration (chronic effects).

**Results:**

As expected from SERCA2a stimulation, compound 8 induced SR Ca^2+^ compartmentalization in STZ myocytes. In-vivo echocardiographic analysis during i.v. infusion and after repeated oral administration of compound 8, detected a significant improvement of diastolic function. Moreover, compound 8 did not affect electrical activity of healthy guinea-pig myocytes, in line with the absence of off-target effects. Finally, compound 8 was well tolerated in mice with no evidence of acute toxicity.

**Conclusions:**

The pharmacological evaluation of compound 8 indicates that it may be a safe and selective drug for a mechanism-based treatment of chronic HF by restoring SERCA2a activity.

**Graphical Abstract:**

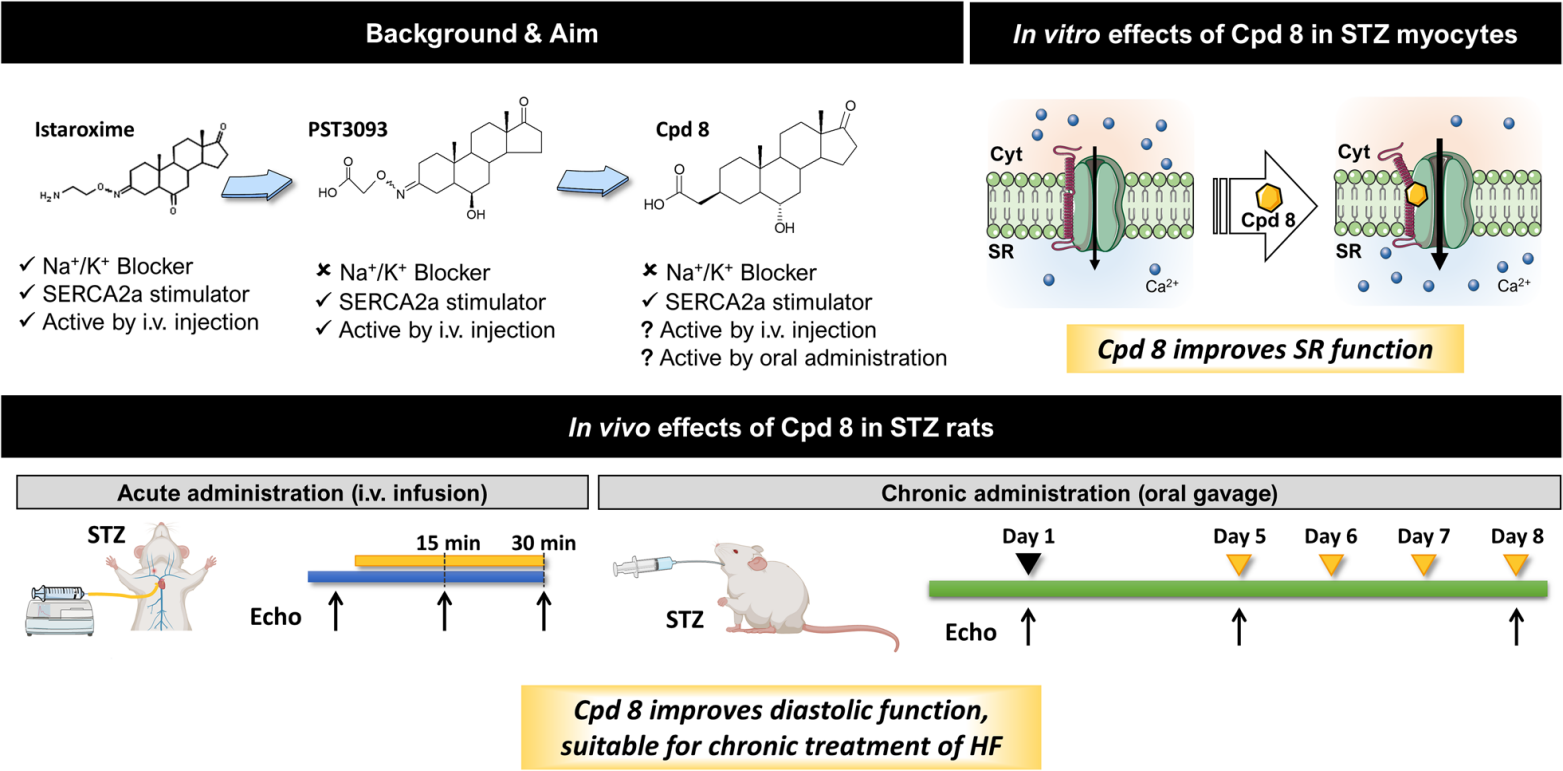

**Supplementary Information:**

The online version contains supplementary material available at 10.1186/s12967-024-04874-9.

## Introduction

Heart failure (HF) has become a global public health burden which affects people worldwide, characterized by a growing incidence of hospitalization and mortality rate. HF with prevailing diastolic dysfunction (HFpEF) is a more recently described entity, for which a specific treatment is still missing. Moreover, dealing with HF in general there is still a critical need of drugs that may improve patient outcomes, without untoward effects [1, 2]. The sarcoplasmic reticulum (SR) Ca^2+^ ATPase (SERCA2a), whose function is usually depressed in HF, is becoming an interesting therapeutic target for HF treatment [3].

Istaroxime is an innovative and unique ino-lusitropic drug that combines the ability to inhibit the Na^+^/K^+^ ATPase and stimulate SERCA2a activity, resulting in improvement of the heart function in healthy and failing animal models and in patients with acute HF (Phase IIb clinical trials) [4–8]. Although endowed with an excellent pharmacodynamic profile, pharmacokinetic studies have indicated that istaroxime has a short plasma half-life (less than 1 h) [6], due to its extensive metabolization to a long-lasting metabolite, PST3093. The latter has a longer half-life (about 9 h), retains the ability to stimulate SERCA2a and it does not inhibit Na^+^/K^+^ ATPase [9]. As a metabolite, PST3093 may in fact contribute to the beneficial effects of istaroxime acutely administered to patients [10]. However, the presence of the potentially genotoxic oxime moiety may limit the chronic usage of both istaroxime and PST3093. This led us to pursue rational design of novel SERCA2a activators based on PST3093 structure, but devoid of the oxime moiety, that would be thus suitable for chronic (oral) treatment of HF [11].

Among the developed PST3093 derivates, compound 8 was one of the two selected compounds showing the ability to recover streptozotocin (STZ)-induced SERCA2a activity depression in a phospholamban (PLN)-dependent manner, analogously to its parent compound PST3093 [11]. We present here the pharmacological evaluation of compound 8 that proves to be a safe and selective SERCA2a stimulator and a favorable drug candidate for chronic HF therapy.

## Materials and methods

The animal study protocols were approved by the Institutional Review Board of Milano Bicocca (29C09.26 and 29C09.N.YRR protocol codes approved in January 2021 and June 2018 respectively) and Chang Gung (CGU107-068 protocol code approved on June 2018) Universities in accordance with the Guide for the Care and Use of Laboratory Animals as adopted and promulgated by the U.S. National Institutes of Health.

Methods are briefly described here; details are given in the Additional section (Additional file 1).

### Disease model

Streptozotocin (STZ)-induced diabetes was selected as a pathological model showing diastolic dysfunction associated to reduced SERCA2a function [12, 13]. Diabetes was induced in Sprague Dawley male rats (150–175 g) by a single i.v. STZ (STZ group, 50 mg/kg in citrate buffer) injection in the tail vein. Control rats (healthy group) received vehicle (citrate buffer). Fasting glycaemia was measured after 1 week and rats with values > 290 mg/dL were considered diabetic [12]. Rats were euthanized by cervical dislocation under anesthesia with ketamine-xylazine (130–7.5 mg kg-1 i.p) 9 weeks after STZ injection.

### Measurements in isolated cardiomyocytes

To ensure stabilization of drug effect, isolated myocytes were analyzed after incubation with compound 8 or vehicle (control) for at least 30 min. The experiments were performed at 35 °C.

The Na^+^/K^+^ ATPase current (I_NaK_) was recorded (V-clamp) in normal rat left ventricular (LV) myocytes as ouabain (1 mM)-sensitive current at − 40 mV, under conditions enhancing I_NaK_ and minimizing contamination by other conductances [12, 14]. I_NaK_ inhibition by compound 8 was expressed as percent reduction of ouabain-sensitive current.

Intracellular Ca^2+^ dynamics were evaluated in Fluo4AM-loaded STZ cardiomyocytes, superfused with Tyrode’s solution. Cytosolic Ca^2+^ was expressed in arbitrary units, i.e. as the ratio between the fluorescence signal and its value during diastole (F/F_0_). Ca^2+^ uptake by the sarcoplasmic reticulum (SR) (proportional to SERCA2a function) was investigated with two protocols addressing SR function under different conditions: post-rest potentiation (PRP) of Ca^2+^ transients (Ca_T_) and “SR Ca^2+^ reloading” after depletion.

The PRP protocol was applied to field-stimulated myocytes as previously shown [12]. Briefly, voltage-induced Ca_T_ were evoked at 2 Hz until steady state Ca_T_ (ssCa_T_) amplitude was achieved. Stimulation was then interrupted for intervals of increasing duration (1-5-10-20 s) and then resumed. Multiple parameters were extracted from the PRP protocol. ssCa_T_ were evaluated in terms of amplitude and decay kinetics (decay t_1/2_). PRP was calculated as the ratio between the first post-rest Ca_T_ (prCa_T_) and the last pre-rest Ca_T_ (ssCa_T_). The prCa_T_/ssCa_T_ ratio provides information on the fate of intracellular Ca^2+^ during the rest interval, largely dictated by the balance between Ca^2+^ extrusion from the cell and Ca^2+^ reuptake into the SR. Also, useful to evaluate this balance is the measurement of SR Ca^2+^ content (Ca_SR_) at various times during the protocol pause. Ca_SR_ was estimated from the amplitude of caffeine (10 mM)-induced Ca_T_, electronically evoked at 0.5 s (CaSR_0.5 s_) and 20 s (CaSR_20s_) following the last stimulated Ca_T_. The ratio CaSR_20s_/CaSR_0.5 s_ was calculated to evaluate post-rest SR Ca^2+^ compartmentalization at rest.

The SR Ca^2+^ reloading protocol (Additional file 1: Figure S1) was designed to examine SR function at multiple levels of Ca^2+^ loading, while eliminating the contribution of the Na^+^/Ca^2+^ exchanger (NCX) to Ca^2+^ clearance. To this end, Ca_T_ and membrane current (I_CaL_) were simultaneously measured in V-clamped myocytes. After SR depletion by a caffeine pulse, Ca_T_ and I_CaL_ were recorded during a reloading pulse train and their features (amplitude, decay t_1/2_) measured at each pulse. The excitation–release (ER) “gain” was calculated as the ratio between Ca_T_ amplitude and Ca^2+^ influx through I_CaL_ up to Ca_T_ peak [5]. During the whole protocol NCX was blocked by Na^+^-free superfusion and Na^+^-free pipette solution.

### Off-target actions

To asses potential off-target effects on ion channels, the effect of compound 8 was evaluated on action potentials (APs), recorded by patch-clamp (I-clamp) from normal guinea-pig LV myocytes. This cell type was selected because of similarity of its AP repolarization to the human one [15]. AP duration at 50% and 90% repolarization (APD_50_ and APD_90_) and diastolic potential (E_diast_) were measured 1) during steady state pacing at several rates, 2) dynamically upon stepping between two rates (to assess APD_90_ adaptation kinetic). During steady state pacing, short-term APD_90_ variability (STV) was calculated from 20–30 subsequent APD_90_ values according to Eq. 1 [16]:

1 $$STV=\sum (|APD(n+1) - APDn|)/[nbeats*\surd 2]$$

The kinetics of APD_90_ adaptation was quantified by estimating the time constant (τ) of the exponential time course of APD_90_ after stepping between two pacing rates.

To further detect potential off-target actions of compound 8, its interaction with a panel of 50 ligands, potentially relevant to off-target effects, was carried out by Eurofins (Taiwan) on crude membrane preparations according to Eurofins procedures. The assays were partly based on radioligand displacement (e.g., for receptors) and partly on spectrophotometric detection of change in function (e.g., for enzymes). Results were compared to appropriate reference standards; a > 50% change in affinity or activity was considered as a positive hit (interaction present). Compound 8 was tested at the concentration of 10 μM.

### In vivo hemodynamic effects in diseased (STZ) rats

In vivo effects of compound 8 were evaluated by echocardiography in STZ rats under urethane (1.25 g/kg, i.p, acute protocol) or ketamine/pentobarbital (60–37.5 mg/kg, i.p., chronic protocol) anesthesia. Studies were carried out during acute (i.v. infusion) and following chronic (oral gavage) treatment.

In the acute protocol, compound 8 or saline (control group) were i.v. infused at 0.2 mg/kg/min (0.16 ml/min); echocardiographic parameters were measured within the same animal before (basal), and at 15 and 30 min of infusion.

In the chronic protocol, compound 8 effects were evaluated following 1 or 4 oral daily administrations (by gavage) at doses of 40 and 80 mg/kg (5 ml/kg body weight) dissolved in saline (Additional file 1: Figure S2). The treatment group was compared to a randomly assigned control group receiving vehicle only. At day 1 all the animals received saline and underwent basal echocardiography. From day 5 to day 8, each group was treated once daily with saline or compound 8 (40 mg/kg or 80 mg/kg); all animals were subjected to echocardiography at day 5 (after 1 dose) and day 8 (after 4 doses). Echo measurements were performed 60 min following gavage.

The following echo indexes were measured, according to the American Society of Echocardiography guidelines [17]: left-ventricular (LV) end-diastolic (LVEDD) and end-systolic (LVESD) diameter, posterior wall thickness (PWT) and interventricular septal thickness (IVST). The Teichholz formula (7/ (2.4 + D) × D^3^, D = linear LV diameter) was used to calculate LV end-diastolic volume (EDV) and end-systolic volume (ESV). Stroke volume (SV) was calculated as the difference between EDV and ESV. LV ejection fraction (EF) was calculated as SV/EDV and expressed in % [18]. Fractional shortening was calculated as FS = (LVEDD-LVESD)*LVEDD^−1^ and expressed in %. Trans-mitral flow velocity was measured (by pulsed Doppler) to obtain early and late filling velocities (E, A waves) and E wave deceleration time (DT). DT was also normalized to E wave amplitude (DT/E ratio). Peak myocardial systolic (s’) and diastolic velocities (e’ and a’) were measured at the mitral annulus by Tissue Doppler Imaging (TDI).

### Drug toxicity in mice

Acute toxicity was determined in the mouse (Albino Swiss CD-1, body weight 30 g). Mice were orally treated, or i.v. injected, with increasing doses of compound 8 to identify the one causing 50% mortality (LD_50_, mg/kg body weight) within 24 h. Compound 8 was dissolved in saline solution and i.v. injected at 50, 100, 200, 300 mg/kg (2–4 animals for each group) or orally administered by gavage at 200 and 700 mg/kg (4 animals for each group). Control animals received vehicle only.

### Statistical analysis

Data are reported as mean ± SEM. Individual means were compared by Student’s *t*-test; multiple means were compared by one-way or two-way ANOVA for repeated measurements (RM), followed by post-hoc Tukey’s multiple comparisons. P < 0.05 was considered as statistically significant in all comparisons. N (number of animals) and n (number of cells) are reported in each figure legend.

## Results

### Chemical structure of compound 8

**Fig. 1:**
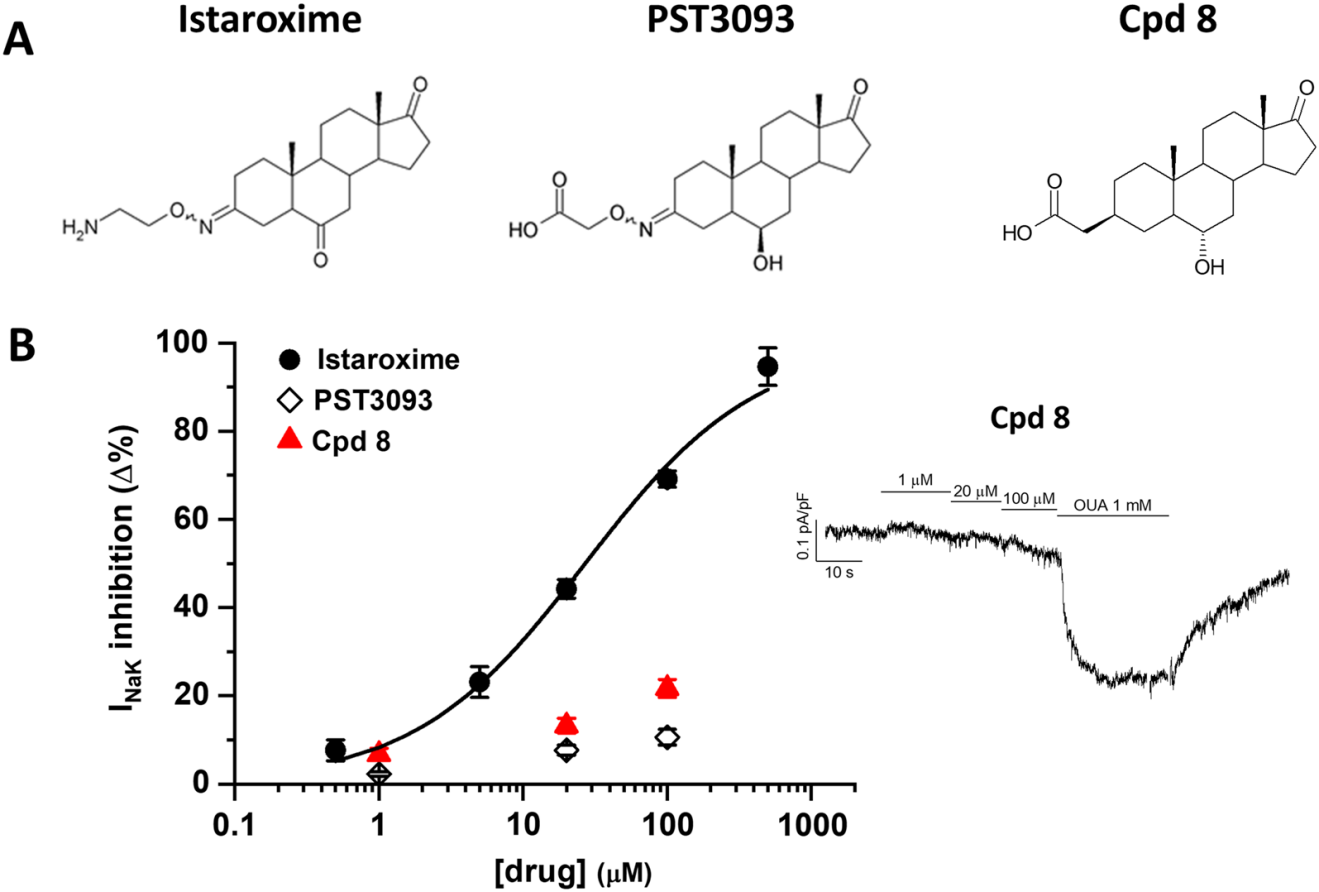
Chemical structure and Na^+^/K^+^ ATPase inhibition. **A** Chemical structures of compound 8 in comparison to istaroxime and its metabolite, PST3093. **B** Concentration–response curve for I_NaK_ inhibition by compound 8 (N = 3, n = 21), in comparison to PST3093 and istaroxime (modified from [12] in rat LV myocytes; I_NaK_ recording under increasing concentration of compound 8 and finally ouabain (OUA as reference) is shown on the right. Data are the mean ± SEM

The chemical structure of compound 8 is shown in Fig. 1A in comparison to that of PST3093 and istaroxime. As previously described [11], PST3093 possesses the 17-androstanone core of istaroxime with a carboxylic acid group instead of the amino group on the C3-oxime linker, and a hydroxyl group with R configuration (or beta-configuration) at C6 instead of istaroxime’s carbonyl. Compound 8 is a PST3093 variant carrying a carboxylic acid group attached through a linker reduced to 2 carbon atoms to the C3 position of the androstane core. Moreover, the oxime double bond in steroid C3 was replaced by a saturated C–C bond in the β-configuration.

### Compound 8 is a selective SERCA2a activator

Compound 8 has been previously characterized in cell-free systems [11] for its stimulatory action on SERCA2a activity, which was achieved at nanomolar concentration, and for its null effect on purified renal Na^+^/K^+^ ATPase up to 10^–4^ M. Such a profile is similar to that of PST3093, its parent compound [9].

Here, we further characterized compound 8 in isolated cardiomyocytes to highlight its selective SERCA2a stimulatory action in the context of intact cells.

The effect on Na^+^/K^+^ ATPase was assessed by the measurement of ouabain-sensitive current (I_NaK_) in normal rat ventricular myocytes. As shown in Fig. 1B, at the limit concentration for solubility (100 µM), compound 8 reduced I_NaK_ by 21.8 ± 2%. While in the same range of that exerted by PST3093 (− 9.2 ± 1.1%) [9], I_NaK_ inhibition by compound 8 was much weaker than observed with istaroxime in the same experimental setting (estimated IC_50_ 32 ± 4 µM) [12].

**Fig. 2:**
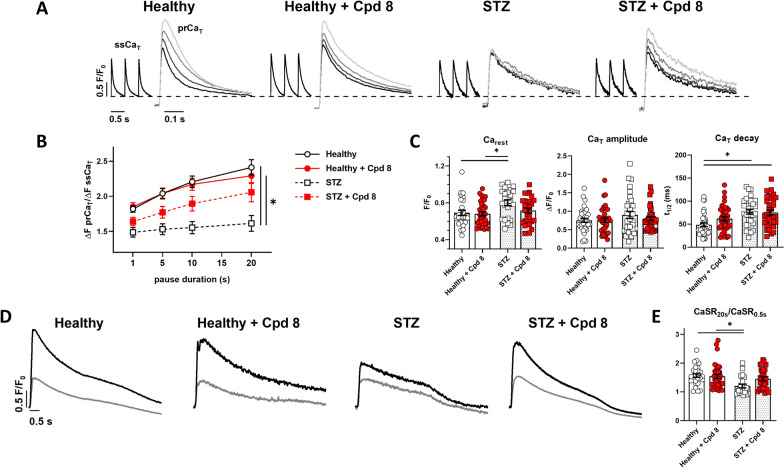
Modulation of SR Ca^2+^ uptake at resting in field stimulated myocytes. **A** Post-rest potentiation (PRP) protocol in Fluo4 field stimulated (2 Hz) myocytes: steady state Ca^2+^ transients (ssCa_T_) and superimposed first post-rest (pr) Ca^2+^ transients (prCa_T_) following increasing resting pause (1-5-10-20 s) are reported in healthy and STZ myocytes, with or w/o 1 μM compound 8. Traces were normalized to own diastolic Ca^2+^ level (dotted line). **B** Analysis of the prCa_T_ amplitude normalized to the amplitude of the pre-pause ssCa_T_ and its pause dependency; healthy N = 3 (n = 34 w/o compound 8, n = 38 with compound 8), STZ N = 3 (n = 31 w/o compound 8, n = 32 with compound 8). *p < 0.05 for the interaction factor in RM two-way ANOVA, indicating a different steepness of curves. **C** Statistics for Ca_rest_, ssCa_T_ amplitude and ssCa_T_ half decay time (t_1/2_); healthy N = 3 (n = 28 w/o compound 8, n = 35 with compound 8), STZ N = 3 (n = 24 w/o compound 8, n = 28 with compound 8). *p < 0.05 (one-way ANOVA plus post-hoc Tukey’s multiple comparison). **D** Caffeine-induced Ca_T_ (Ca_SR_) in field stimulated healthy and STZ myocytes with or w/o 1 μM compound 8 following 0.5 s (in grey) or 20 s (in black) resting pause. **E** Statistics for CaSR_20s_/CaSR_0.5 s_; healthy N = 3 (n = 28 w/o compound 8, n = 35 with compound 8), STZ N = 3 (n = 23 w/o compound 8, n = 28 with compound 8). *p < 0.05 (one way ANOVA plus post-hoc Tukey’s multiple comparison)

The effect of compound 8 (1 µM) on SERCA2a was assessed in myocytes isolated from healthy and STZ-rats. A comprehensive in vivo and in vitro analysis of STZ model is reported in previous studies of our group [9, 11, 12]. To evaluate the SR ability to accumulate Ca^2+^ during rest, we used the PRP protocol. As shown in Fig. 2A, B, following increasing resting pauses, the amplitude of the prCa_T_ increased progressively in healthy myocytes; PRP was depressed in STZ myocytes at all resting intervals, as expected from STZ-induced SERCA2a down-regulation [12]. Compound 8 (1 µM) failed to affect PRP in healthy myocytes, while it increased it toward normal values in STZ myocytes (Fig. 2B).

PRP is relevant to the present study because it may reflect Ca^2+^ compartmentalization within the SR during rest. To validate this postulate, we measured cytosolic Ca^2+^ at the end of 20 s rest interval (Ca_rest_), and the ratio between SR Ca^2+^ contents after long (20 s) and short (0.5 s) rest intervals respectively (CaSR_20s_/CaSR_0.5 s_, see Methods). Consistent with SERCA2a depression, in STZ myocytes Ca_rest_ was increased and the CaSR_20s_/CaSR_0.5 s_ ratio was decreased as compared to healthy myocytes. Albeit statistical significance was not achieved, compound 8 tended to restore these variables toward their healthy value in STZ myocytes, but it was ineffective in healthy ones (Fig. 2C–E). Taken together, these results suggest that compound 8 improved Ca^2+^ sequestration into the SR during the post-train quiescence period, particularly under conditions of SERCA2a depression.

ssCa_T_ amplitude was not significantly changed in STZ myocytes as compared to healthy ones, and it was not modified by compound 8. Albeit ssCa_T_ decay kinetic was slower in STZ myocytes, compound 8 failed to change it significantly (Fig. 2C). Although apparently in contrast with SERCA2a activation, these was true also for the parent compounds istaroxime [12] and PST3093 [9] in the same experimental setting and probably due to the large variance of these parameters when measured during the action potential (i.e. under field-stimulation) in the presence of disease-induced changes in electrical activity [12].

**Fig. 3:**
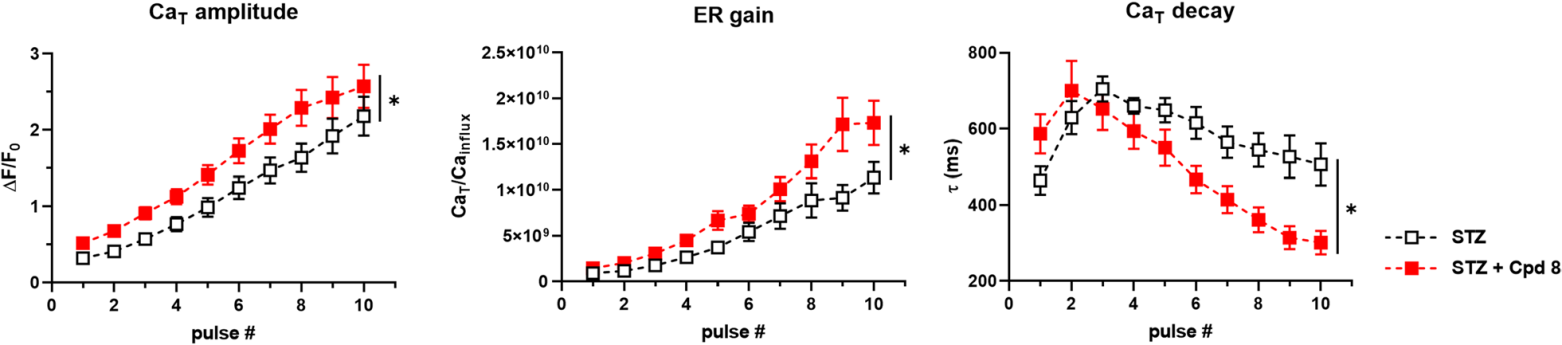
Modulation of SR Ca^2+^ uptake under NCX inhibition in V-clamped STZ myocytes. SR Ca^2+^ loading by a train of V-clamped pulses was initiated after caffeine-induced SR depletion; NCX was blocked by Na^+^ substitution to identify SERCA2a-specific effects (see Methods and Additional file 1: Figure S1). Panels from left to right: Ca_T_ amplitude, excitation-release (ER) gain (the ratio between Ca_T_ amplitude and Ca^2+^ influx through I_CaL_), time constant (τ) of Ca_T_ decay; STZ N = 3 (n = 18 w/o compound 8, n = 23 with compound 8). *p < 0.05 for the interaction factor in RM two-way ANOVA, indicating a different steepness of curves

To assess SR Ca^2+^ uptake under more controlled conditions, emphasizing SERCA2a role (NCX inhibition), compound 8 effect was analyzed under V-clamp, by using the “SR loading” protocol (see Methods and Additional file 1: Figure S1). As previously shown [9], all the parameters measured by this protocol allow functional appreciation of SERCA2a downregulation in failing myocytes. Here we tested compound 8 (1 µM) effects in STZ myocytes (Fig. 3). Compound 8 sharply accelerated Ca_T_ decay and increased the rate at which Ca_T_ amplitude and ER-gain increased over the reloading train. Comparable results have been obtained by istaroxime at a concentration marginally affecting Na^+^/K^+^ ATPase [12], by its metabolite PST3093 [9] and another PST3093 derivative [11]. Overall, compound 8, as its parent compounds, restored SR function in failing myocytes, i.e. in the context of a pathological cellular environment; these effects are suggestive of the ability of the compound to stimulate SERCA2a activity.

### Compound 8 does not show off-target effects

**Fig. 4:**
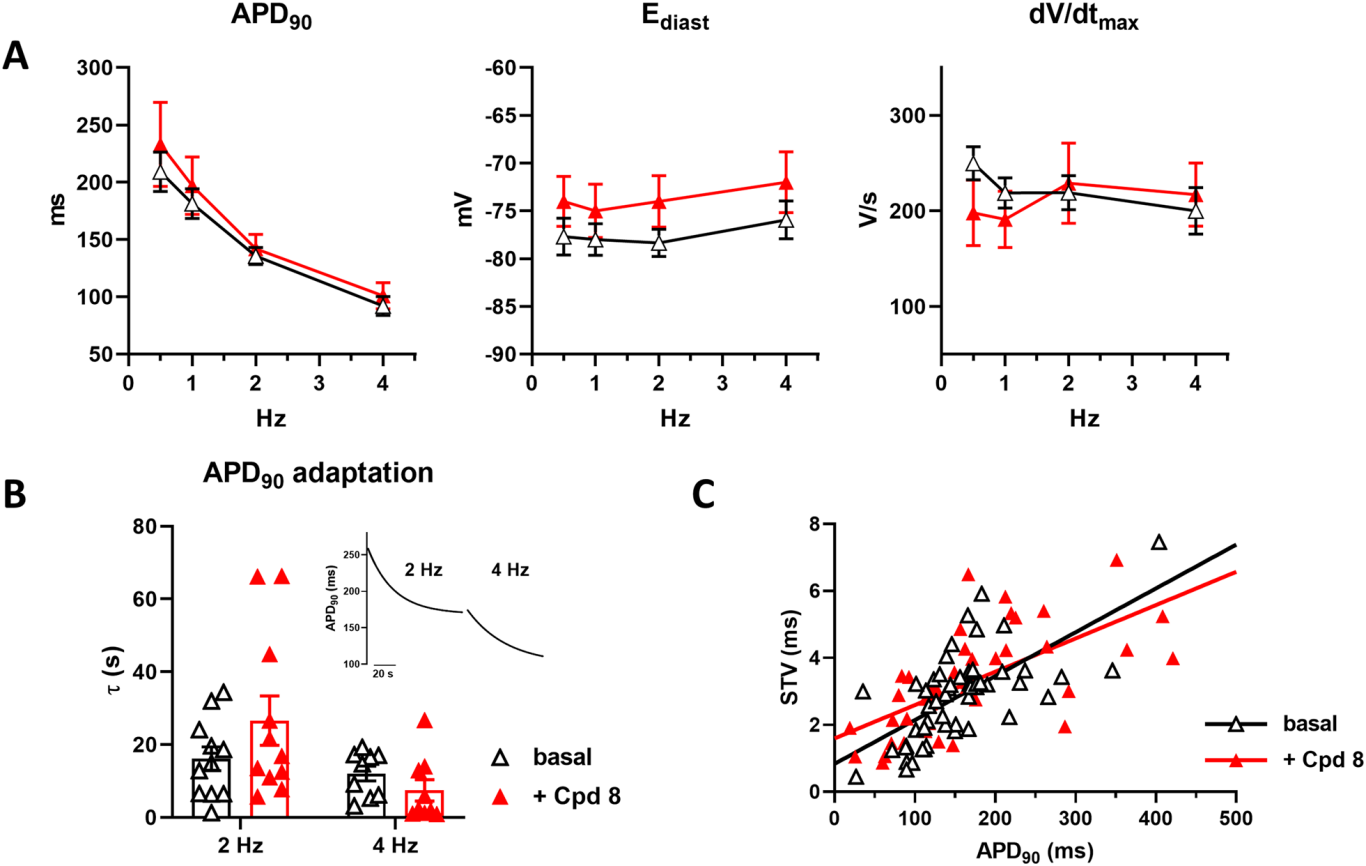
Modulation of electrical activity in healthy guinea pig myocytes. The effect of 1 μM compound 8 was tested on action potential (AP) parameters and their steady-state rate dependency in guinea pig myocytes. **A** Effect on the rate dependency of AP duration (APD_50_ and APD_90_), diastolic potential (E_diast_) and maximal phase 0 velocity (dV/dt_max_); n = 13 w/o compound 8, n = 11 with compound 8. **B** Effect on the time constant (τ) of APD_90_ adaptation following a step change in rate. 4 Hz: n = 9 w/o compound 8, n = 10 with compound 8; 2 Hz: n = 11 with or w/o compound 8. **C** Effect on the correlation between STV of APD_90_ and APD_90_ values; data from 1, 2 and 4 Hz were pooled

To assess the electrophysiological safety of compound 8, its effects on AP of LV myocytes were investigated. Guinea pig myocytes were used because in this species repolarization is closer to the human one. Compound 8, tested at the concentration of 1 μM, did not change AP parameters (APD_90_, E_diast_, dV/dt_max_) (Fig. 4A). Notably, also APD rate-dependency at steady-state and the kinetics of APD adaptation following a step change in rate were unaffected by the agent (Fig. 4B). STV of APD_90_, a reporter of repolarization instability, was also unaffected by compound 8 at all pacing rates (Fig. 4C). Similar results were also obtained with compound 8 at lower concentrations (data not shown).

The off-target effects of 10 μM compound 8 were also analysed on a broader set of targets by a high-throughput interaction analysis. Molecular targets other than SERCA2a (50 items), including membrane receptors, key enzymes, ion channels and transporters, are shown in Additional file 1: Table S1. None among the 50 items met criteria for significance of interaction. Thus, at least for the ligands shown in Additional file 1: Table S1, no off-target action of compound 8 is expected. Similar findings were observed with the parent compound PST3093 [19].

### Compound 8 is well tolerated in vivo

Compound 8 acute toxicity was preliminarily evaluated in CD1 mice following i.v. and oral administration. The compound was well tolerated and did not cause death up to 300 mg/kg after i.v. administration, similarly to PST3093 [9]. For comparison, istaroxime LD_50_ following i.v. infusion was 23 mg/kg [9]. By oral administration, compound 8 did not cause death up to 700 mg/kg, the highest tested dose. For comparison, deaths were observed with oral istaroxime at 200 mg/kg [11], thus emphasizing suitability of compound 8 for chronic oral treatment.

### Compound 8 is active following acute and chronic in vivo treatment

The effect of compound 8 on echocardiographic indexes in the STZ cardiomyopathic rat model was assessed after i.v. infusion or oral administration. In a previous study of our group [9], STZ model was extensively characterized through echocardiographic analysis. In comparison to healthy controls, STZ rats showed diastolic function impairment. Indeed, early filling velocity (E) and TDI relaxation velocity (e’) were reduced and the deceleration time (DT)/E ratio was increased. Systolic function was more mildly affected, as reported by small changes in fractional shortening (FS), ejection fraction (EF) and TDI contraction velocity (s’) [9].

#### Acute effects (i.v. administration)

**Fig. 5:**
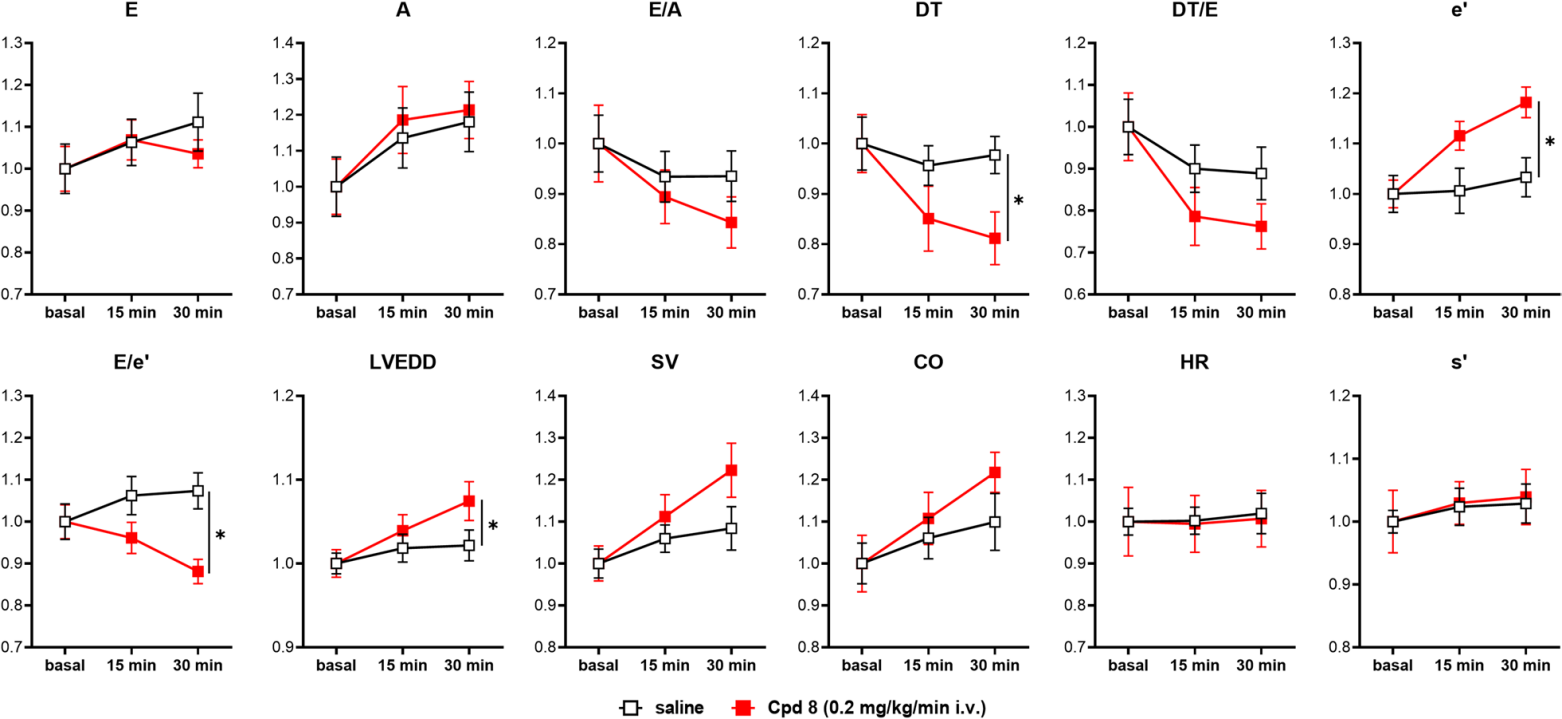
In vivo effects during i.v. infusion in STZ rats. Compound 8 or vehicle (saline) was i.v. infused at 0.2 mg/kg/min under urethane anesthesia, in rats 8 weeks after STZ treatment. Echocardiographic parameters (see also Additional file 1: Figure S3) were measured under basal condition, at 15 and 30 min during drug infusion. Data are mean ± SEM. Saline group N = 8, compound 8 group N = 11; *p < 0.05 vs saline group for the interaction factor in RM two-way ANOVA. E, A: early and late mitral inflow velocities; *DT* E wave deceleration time; *e’* early diastolic mitral annulus velocity; *LVEDD* LV end diastolic diameter; *SV* stroke volume; *CO* cardiac output; *HR* heart rate: *s’* peak systolic tissue velocity

Compound 8 or its vehicle (saline) were i.v. infused in STZ rats at the rate of 0.2 mg/kg/min under urethane anesthesia; echocardiographic parameters were measured at 15 and 30 min of infusion (Fig. 5 and Additional file 1: Figure S3, absolute values of each echo parameter are shown in Additional file 1: Table S2). Compound 8 effects over time were assessed by comparison with vehicle ones, i.e. by testing significance of the interaction factor (time x treatment) between the two treatment groups in 2-way ANOVA. Based on this analysis, statistically significant effects of compound 8 included a decrease in DT, an increment in tissue relaxation velocity (e’) (resulting in an increase of e’/a’ and in a decline of E/e’) and an increase in LVEDD, all parameters reflecting improved diastolic function. Albeit without achievement of statistical significance, a trend to improvement was also visible for indexes of overall cardiac function, such as SV and CO. On the other hand, heart rate (HR) and systolic indexes, such as systolic tissue velocity (s’) and FS (Additional file 1: Figure S3) were not significantly affected by compound 8; a significant reduction in IVST in systole and diastole with a small increase of LVESD was also observed (Additional file 1: Figure S3). Most drug effects tended to level at 15 min of infusion.

#### Chronic effects (oral administration)

**Fig. 6:**
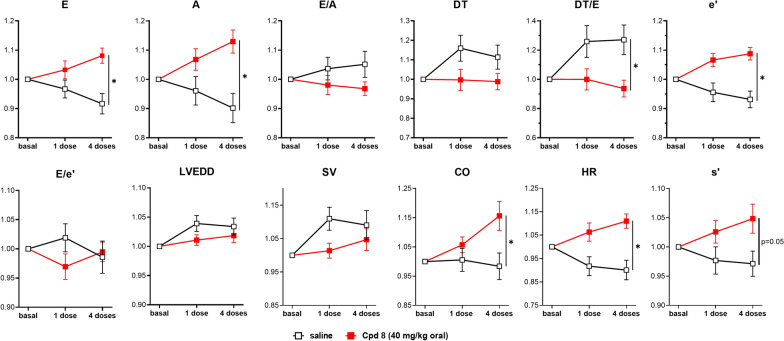
In vivo effects following oral treatment in STZ rats (40 mg/kg compound 8). Rats were treated with 1 or 4 oral daily doses of compound 8 (40 mg/kg) or saline, accordingly to the protocol shown in Additional file 1: Figure S2. Echocardiographic parameters (see also Additional file 1: Figure S4) were measured in each group 60-min post treatment under ketamine/pentobarbital anesthesia; each measurement was normalized to its basal value to highlight changes between experimental groups (saline and 40 mg/kg compound 8). Data are mean ± SEM; saline N = 21, 40 mg/kg compound 8 N = 22. *p < 0.05 vs saline group for the interaction factor in RM two-way ANOVA

**Fig. 7:**
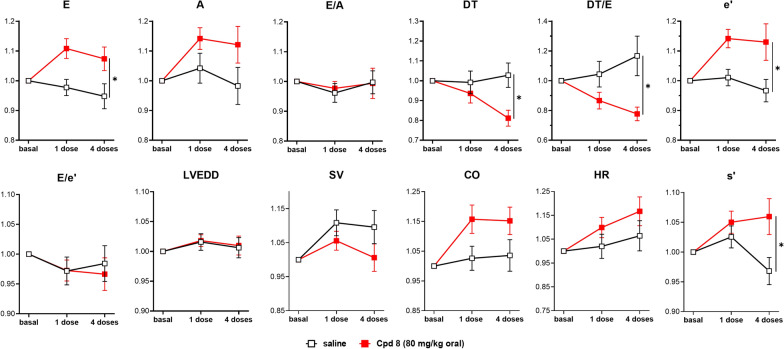
In vivo effects following oral treatment in STZ rats (80 mg/kg compound 8). Rats were treated with 1 or 4 oral daily doses of compound 8 (80 mg/kg) or saline, accordingly to the protocol shown in Additional file 1: Figure S2. Echocardiographic parameters (see also Additional file 1: Figure S5) were measured in each group 60-min post treatment under ketamine/pentobarbital anesthesia; each measurement was normalized to its basal value to highlight changes between experimental groups (saline and 80 mg/kg compound 8). Data are mean ± SEM; saline N = 19, 80 mg/kg compound 8 N = 21. *p < 0.05 vs saline group for the interaction factor in RM two-way ANOVA

For the oral protocol, STZ rats were administered with single or repeated (4) doses of compound 8 (40 or 80 mg/kg) or saline (Figs. 6, 7 and Additional file 1: Figures S4-S5, absolute values of each echo parameter are shown in Additional file 1: Tables S3-S4) under ketamine/pentobarbital anesthesia. To facilitate the comparisons among compound doses and treatment duration, each echo parameter measured at day 5 (after a single dose) and day 8 (after four doses) was normalized to its basal value measured at day 1 (protocol outline in Additional file 1: Figure S2).

While some among compound 8 effects were already detected after a single 40 mg/kg dose, 4 repeated administrations had a clear-cut incremental effect (Fig. 6 and Additional file 1: Figure S4). The specific parameters affected were somewhat different from those modified by acute i.v. administration (previous section), including both diastolic (E, e’, DT/e’) and some systolic (A, a’, s’) indexes. Notably, the simultaneous increase in E and A waves, expected from concomitant lusitropy and inotropy, left the E/A and e’/a’ ratios unchanged. On the other hand, compound 8 failed to affect other systolic parameters, such as FS, SV and EF. CO was significantly increased by the compound, but a concomitant increment in HR (possibly accounting for the blunted response of SV) complicates the interpretation of this change.

At the higher dose (80 mg/kg, Fig. 7 and Additional file 1: Figure S5) compound 8 significantly changed several diastolic indexes already after a single administration, with little additional effect provided by repeated administration. In qualitative terms, the effects of the 80 mg/kg dose were similar to those observed with the 40 mg/kg one.

Overall, the effects of oral administration of compound 8 are in line with those of i.v. administration to indicate improvement of cardiac hemodynamics. Notably, even though at the lower dose, oral administration of compound 8 differed from i.v. one for producing a significant increment of HR. The mechanism for this difference, of potential clinical relevance, may deserve further investigation. Cumulative effect of repeated administration was clearly observed at the lower oral dosage.

## Discussion

The present study summarizes the preclinical data concerning compound 8, a derivative of PST3093, the long-lasting istaroxime metabolite. Compound 8 is devoid of the oxime moiety and was selected in an in vitro screening based on optimization of selectivity for SERCA2a activation vs inhibition of the Na^+^/K^+^ ATPase [11]. The emerging profile of compound 8 indicates that it has a low acute toxicity, it is active in isolated myocytes and in reversing STZ-induced diastolic dysfunction in vivo. Compound 8 effects were qualitatively similar after i.v. and oral administration; incremental effect during repeated once-a-day dosing suggests pharmacokinetics suitable for chronic usage.

Previous results on molecular function in cell free systems [11] indicate that compound 8 enhances SERCA2a enzymatic activity selectively, i.e. without appreciably affecting Na^+^/K^+^ pump activity. The present studies at cellular and in vivo levels, confirm this view and collectively point to compound 8 ability to improve Ca^2+^ confinement within the SR. This extends the conclusions of previous molecular studies to the fully integrated biological system.

The SERCA2a stimulatory activity of istaroxime and PST3093, from which compound 8 was derived, depends on the presence of PLN [9, 20]; thus, suggesting that these compounds enhance SERCA2a activity by relieving its inhibition by PLN. The same likely applies to compound 8 [11], which has a closely similar chemical structure. Hence, these compounds can be collectively defined as “PLN antagonists”, a novel class of drug action.

A diseased heart model (the STZ diabetic rat) was chosen for in vivo studies; this is justified by previous observations with the parent compounds. PLN antagonism by istaroxime, the prototype PLN antagonist, was firstly detected in healthy guinea-pig myocytes [5] and reproduced in murine ones [21]. However, istaroxime effect was substantially enhanced in failing guinea-pig preparations [22]. Rat myocytes, best suited for in vivo studies, are relatively insensitive to PLN antagonism when healthy, to become responsive when SERCA2a activity is depressed by disease. This is the case for STZ-induced diabetes, in which consistent with primarily diastolic dysfunction in clinical diabetes, myocytes are characterized by SERCA2a down-regulation [9, 12]. These considerations, as well as the added translational value provided by relevance to human pathology, led us to adopt the STZ (diabetic) rat as experimental model. Why the effect of PLN antagonism becomes more apparent whenever baseline SERCA2a function is diminished is a matter of speculation, an interesting one, but of limited translational relevance for an agent meant to treat diseased hearts.

Some among the present results were unexpected based on PLN antagonism and deserve to be separately discussed. While SERCA2a stimulation is expected to increase the rate of decay of V-triggered Ca_T_, compound 8 failed to change Ca_T_ decay kinetic measured in field-stimulated myocytes (during electrical activity) (Fig. 2C), in which all Ca^2+^ transports were intact. On the other hand, compound 8 sharply decreased $\tau_{decay}$ in the “reloading protocol” (Fig. 3) in V-clamped cells, in which SR uptake function largely depends on SERCA2a only. This apparent discrepancy might be attributed to the variability of the rate of Ca_T_ decay in field stimulated cells in comparison to the same parameter measured controlling membrane potential (V-clamp), or to NCX contribution to diastolic Ca^2+^ clearance. Notably, the same was true for PST3093, which is nonetheless endowed with clear-cut lusitropic effect in vivo [9].

Acute i.v. infusion of compound 8 (Fig. 5) improved diastolic relaxation in STZ rats. Similar results on diastolic indexes were obtained following four oral daily doses at 40 mg/kg and a single dose at 80 mg/kg. This argues against significant changes in SERCA2a-modulating effect by liver metabolism.

Among the main goals of this study was the evaluation of chronic in vivo effects of orally administered compound 8. In vivo data published so far support the high therapeutic potential of istaroxime [6–8, 10], its metabolite PST3093 [9] and follow-on derivatives [11]. Nonetheless, this is the first study evaluating chronic effects of the lead follow-on compound through its oral administration in a disease model. Accumulation of effect over repeated dosing every 24 h points to a relatively slow clearance of the compound. Accordingly, while compound 8 pharmacokinetics is still unknown, its chemical structure predicts a plasma half-life comparable to that of PST3093 (about 9 h in humans) [9]. Accumulation of effects was seen with the higher dosage of compound 8 only in DT reduction, likely because of early achievement of saturating plasma levels.

Effects of compound 8 on systolic indexes were marginal; indeed, among systolic indexes, only s’ parameter significantly increased after 4 oral doses (Figs. 6–7). HR was significantly affected by the compound after the lowest oral dose (40 mg/kg), but this effect was not clearly detected at the highest oral dose (80 mg/kg) and after i.v. infusion. Moreover, since effects on HR were absent when the compound was i.v. infused, further investigations are necessary to clarify this point. Nonetheless, a comprehensive characterization of the underlying mechanisms is beyond the scope of the current study.

A pure SERCA2a activator might exert substantial antiarrhythmic effects by inhibiting Ca^2+^ waves [23, 24], at least under the common conditions characterized by SR instability (e.g. HF). Further, focused studies are necessary to better characterize the potential antiarrhythmic effects of SERCA2a stimulators. Furthermore, SR Ca^2+^ compartmentalization has potential long-term effects on energetic efficiency and biology of cardiac myocytes [23]. Among them, higher SR Ca^2+^ content might conceivably support Ca^2+^ transfer to mitochondria. However, we have recently found that a PLN mutation resulting in SERCA2a enhancement is associated with depression of mitochondrial function instead [25]. Even if it is difficult to rule out off-target effects of mutant PLN, this observation may discourage from predicting the relationship between SERCA2a function and mitochondrial respiration by simple reasoning. Nonetheless, evaluation of the effect of SERCA2a stimulators on mitochondrial function may be worthwhile.

In summary, the specific lusitropic effect of compound 8 in STZ rats, detected both during i.v. infusion and after oral administration, can be attributed to recovery of SERCA2a function. Compound 8 represents the first small-molecule SERCA2a activator that can be considered for oral administration as a chronic treatment of HF based on such an innovative mechanism of action.

## Study limitations

A limitation of echocardiografic studies in small animals is the intrinsic variability of measurements, which may explain why, albeit consistent in pointing to improvement of diastolic function, the parameters affected by the different administration protocols did not coincide. Echocardiography in rodents is also encumbered by the potentially confounding effect of anesthesia, which is nonetheless required for the procedure and necessarily different in i.v. infused and orally treated animals.

In translating the present data to the clinical setting, potential pathophysiological differences between the STZ rat model and clinical HF should be considered. Indeed, particularly conspicuous changes in body fluids, sympathetic nervous activity and HR characterize the STZ animal model, which may impact on cardiac function independently from the changes in cellular Ca^2+^ handling [26].

### Supplementary Information

**Additional file 1: Figure S1.** Protocol to evaluate intracellular Ca2+ dynamics in patch-clamped cells under Na+ free condition. **Figure S2.** Protocol outline for oral treatment of STZ rats with compound 8 at 40 mg/kg or 80 mg/kg vs control group (saline). **Figure S3.** *In vivo *effects during i.v. infusion in STZ rats. **Figure S4.** *In vivo *effects following oral treatment in STZ rats (40mg/kg compound 8). **Figure S5.** *In vivo *effects following oral treatment in STZ rats (80mg/kg compound 8). **Table S1.** Effect of compound 8 (10 μM) on the panel of molecular targets. **Table S2, S3, S4.** Echocardiographic and tissue Doppler parameters in STZ rats (raw data).

## Data Availability

Data are available from the corresponding author upon reasonable request.

## Abbreviations

a’: Late diastolic mitral annulus velocity
A: Late diastolic mitral inflow velocity
AP: Action potential
APD: Action potential duration
APD_50_ and APD_90_: AP duration at 50% and 90% of repolarization
Ca_rest_: Resting Ca^2+^ at 20 s pause end
Ca_SR_: Sarcoplasmic reticulum Ca^2+^ content
Ca_T_: Ca^2+^ transient
CO: Cardiac output
Cpd: Compound
DT: Deceleration time
e’: Early diastolic mitral annulus velocity
E: Early diastolic mitral inflow velocity
E_diast_: Diastolic potential
EDV: End diastolic volume
EF: Ejection fraction
ER: Excitation-release
ESV: End systolic volume
F_0_: Reference fluorescence
FS: Fractional shortening
HF: Heart failure
HR: Heart rate
I_CaL_: L type Ca^2+^ current
I_NaK_: Na^+^/K^+^ ATPase current
i.p.: Intraperitoneal administration
i.v.: Intravenous administration
IVSTd or IVSTs: Interventricular septum thickness in diastole or systole
LD_50_: in vivo Dose causing 50% mortality
LV: Left ventricle
LVEDD or LVESD: LV end-diastolic or systolic diameter
NCX: Na^+^/Ca^2+^ exchanger
PLN: Phospholamban
PRP: Post-rest potentiation
PWTd or PWTs: Posterior wall thickness in diastole or systole
SERCA: Sarcoplasmic reticulum Ca^2+^ ATPase
SR: Sarcoplasmic reticulum
STV: Short term variability
STZ: Streptozotocin
SV: Stroke volume
s’: Peak systolic tissue velocity
t_1/2_: Ca^2+^ transient decay half-time
τ_decay_: Time-constant of Ca_T_ decay
TDI: Tissue Doppler Imaging
