# Supplementary material for: Selective SERCA2a activator as a candidate for chronic heart failure therapy
Source: J Transl Med. 2024 Jan 19;22:77. doi: 10.1186/s12967-024-04874-9 (PMC10797746; doi:10.1186/s12967-024-04874-9)
Supplement: Supplementary file 1 — Additional file 1: Figure S1. Protocol to evaluate intracellular Ca2+ dynamics in patch-clamped cells under Na+ free condition. Figure S2. Protocol outline for oral treatment of STZ rats with compound 8 at 40 mg/kg or 80 mg/kg vs control group (saline). Figure S3. In vivo effects during i.v. infusion in STZ rats. Figure S4. In vivo effects following oral treatment in STZ rats (40mg/kg compound 8). Figure S5. In vivo effects following oral treatment in STZ rats (80mg/kg compound 8). Table S1. Effect of compound 8 (10 μM) on the panel of molecular targets. Table S2, S3, S4. Echocardiographic and tissue Doppler parameters in STZ rats (raw data). [file 12967_2024_4874_MOESM1_ESM.pdf]

SUPPORTING INFORMATION

"Selective SERCA2a activator as a candidate for chronic heart failure therapy" by Arici M & Shih-Che Hsu et al.

Index

Supplementary Methods pp. 2-3

Supplementary Figures and Tables: pp. 4-13

Figure S1: Protocol to evaluate intracellular Ca^{2+} dynamics in patch-clamped cells under Na^{+} free condition; p. 4
Figure S2: Protocol outline for oral treatment of STZ rats with compound 8 at 40 mg/kg or 80 mg/kg vs control group (saline); p. 5
Figure S3: *In vivo* effects during i.v. infusion in STZ rats; p. 6
Figure S4: *In vivo* effects following oral treatment in STZ rats (40mg/kg compound 8); p. 7
Figure S5: *In vivo* effects following oral treatment in STZ rats (80mg/kg compound 8). p. 8

Table S1: Effect of compound 8 (10 μM) on the panel of molecular targets; pp. 9-10
Table S2-S3-S4: Echocardiographic and tissue Doppler parameters in STZ rats (raw data). p. 11-13

References p. 14

Supplementary Methods

Animal models

Male Sprague Dawley (SD) rats (150-175 gr) were used to generate STZ-induced diabetic cardiomyopathy model to test compounds *in vivo* and *in vitro*; female Dunkin-Hartley guinea pigs (175-200 g) were used for I-clamp measurements in ventricular myocytes and finally, male Albino Swiss CD1 mice (30 g) were used for acute *in vivo* toxicity.

In-vitro effects for ligands potentially accounting for off-target actions

Analysis of compound 8 interaction with a panel of 50 ligands was carried out by Eurofins (Taiwan) on crude membrane preparations according to Eurofins described procedures. The assay is partly based on radioligand displacement (e.g. for receptors) and partly on spectrophotometric detection of change in function (e.g. for enzymes). Results were compared to appropriate reference standards; a >50% change in affinity or activity was considered as a positive hit (interaction present).

Measurements in isolated ventricular myocytes

Rat and guinea-pig LV ventricular myocytes were isolated by using a retrograde coronary perfusion method previously published (Rocchetti et al., 2003) with minor modifications. Rod-shaped, Ca^{2+}-tolerant myocytes were used within 12 h from dissociation. LV myocytes were clamped in the whole-cell configuration (Axopatch 200A, Axon Instruments Inc., Union City, CA). During measurements, myocytes were superfused at 2 ml/min with Tyrode's solution containing 154 mM NaCl, 4 mM KCl, 2 mM $CaCl_2$, 1 mM $MgCl_2$, 5 mM HEPES/NaOH, and 5.5 mM D-glucose, adjusted to pH 7.35. A thermostated manifold, allowing for fast (electronically timed) solution switch, was used for cell superfusion. All measurements were performed at 35 °C. The pipette solution contained 110 mM K^+ -aspartate, 23 mM KCl, 0.2 mM $CaCl_2$ (10^{-7} M calculated free-Ca^{2+} concentration), 3 mM $MgCl_2$, 5 mM HEPES-KOH, 0.5 mM EGTA-KOH, 0.4 mM GTP-Na^+ salt, 5 mM ATP-Na^+ salt, and 5 mM creatine phosphate Na^+ salt, pH 7.3. Membrane capacitance and series resistance were measured in every cell but left uncompensated. Current signals were filtered at 2 KHz and digitized at 5 KHz (Axon Digidata 1200). Trace acquisition and analysis was controlled by dedicated software (Axon pClamp 8.0).

<u>Na^+/K^+ ATPase current (I_{NaK}) measurements.</u> I_{NaK} was recorded in isolated rat LV myocytes (Rocchetti et al., 2003; Alemanni et al., 2011) as the holding current recorded at -40 mV in the presence of Ni^{2+} (5 mM), nifedipine (5 µM), Ba^{2+} (1 mM) and 4-aminopyridine (2 mM) to minimize contamination by changes in Na^+/Ca^{2+} exchanger (NCX), Ca^{2+} and K^+ currents, respectively. Tetraethylammonium-Cl (20 mM) and EGTA (5 mM) were added to the pipette solution and intracellular K^+ was replaced by Cs^+. To optimize the recording conditions, I_{NaK} was enhanced by increasing intracellular Na^+ (10 mM) and extracellular K^+ (5.4 mM). All drugs were dissolved in dimethyl sulfoxide (DMSO). Control and test solutions contained maximum 1:100 DMSO.

<u>Intracellular Ca^{2+} dynamics.</u> LV myocytes were incubated in Tyrode's solution for 30 min with the membrane-permeant form of the dye, Fluo4-AM (10 µM), and then washed for 15 min to allow dye de-esterification. Fluo4 emission was collected through a 535 nm band pass filter, converted to voltage, low-pass filtered (100 Hz) and digitized at 2 kHz after further low-pass digital filtering (FFT, 50 Hz). After subtraction of background luminescence, a reference fluorescence (F_0) value was used for signal normalization (F/F_0). Cytosolic Ca^{2+} activity was dynamically measured in field stimulated (2 Hz) and

patch-clamped rat LV myocytes. In the first case, fluorescence in diastole was used as F_0 for signal normalization (F/F_0).

In patch-clamped myocytes membrane current, whose time-dependent component mainly reflected the sarcolemmal Ca^{2+} current (I_{CaL}), was simultaneously recorded. Drug effects on SR Ca^{2+} uptake rate were evaluated with a "SR loading protocol" specifically devised to rule out the contribution of NCX and to assess the SR Ca^{2+} uptake rate at multiple levels of SR Ca^{2+} loading (Rocchetti et al., 2005) (protocol in Figure S1). The protocol consisted in emptying the SR by a brief caffeine (10 mM) pulse and then progressively refilling it by 7-10 voltage steps (-35 to 0 mV) activating Ca^{2+} influx through I_{CaL}. NCX was blocked by omission of Na^+ from intracellular and extracellular (replaced by equimolar Li^+ and 1 mM EGTA) solutions. The procedure is in agreement with published methods, with minor modifications (Rocchetti et al., 2005; Alemanni et al., 2011; Torre et al., 2022). Multiple parameters, suitable to quantify SR Ca^{2+} uptake, can be extracted from Ca^{2+} and I_{CaL} response to the protocol: the time constant (τ) of cytosolic Ca^{2+} decay within each V-step largely reflects net Ca^{2+} flux across the SR membrane (the faster SR Ca^{2+} uptake, the smaller τ decay). Because of the steep dependency of Ca_T amplitude on SR Ca^{2+} content, the rate at which Ca_T amplitude increases across the subsequent pulses of the protocol reflects the rate at which the SR refills. To rule out the potential contribution of changes in I_{CaL}, in each loading step, Ca_T amplitude was normalized to Ca^{2+} influx (estimated from I_{CaL} integral up to Ca_T peak) to obtain excitation-release (ER) gain. As expected from its strong dependency on SR Ca^{2+} content, this parameter progressively increases during the loading protocol. Diastolic Ca^{2+} of the first step was used as F_0 for signal normalization (F/F_0). Specificity of the "loading protocol" parameters in detecting SERCA2a activation is supported by the observation that they did not detect any effect of digoxin, an inotropic agent blocking the Na^+/K^+ pump and devoid of SERCA2a stimulating effect (Rocchetti et al., 2005; Alemanni et al., 2011).

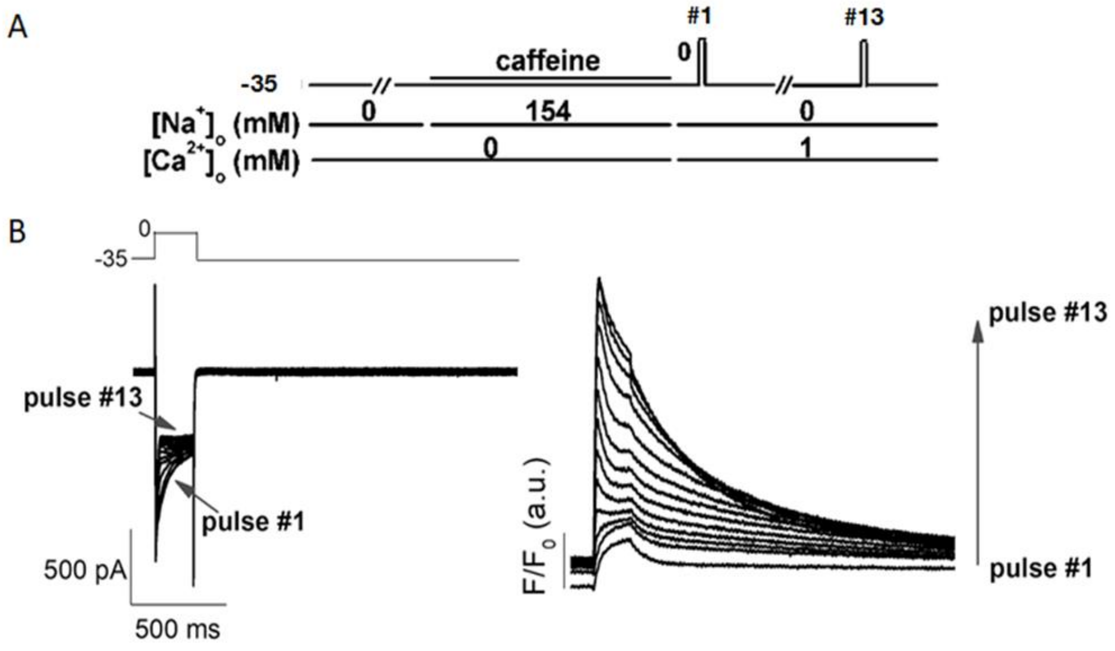

Figure S1. Protocol to evaluate intracellular Ca^{2+} dynamics in patch-clamped cells under Na^{+} free condition. **A)** Protocol outline. **B)** Transmembrane current (left) and Ca^{2+} transients (right) recordings during SR reloading after caffeine-induced SR depletion in patch-clamped cells. See Methods for details.

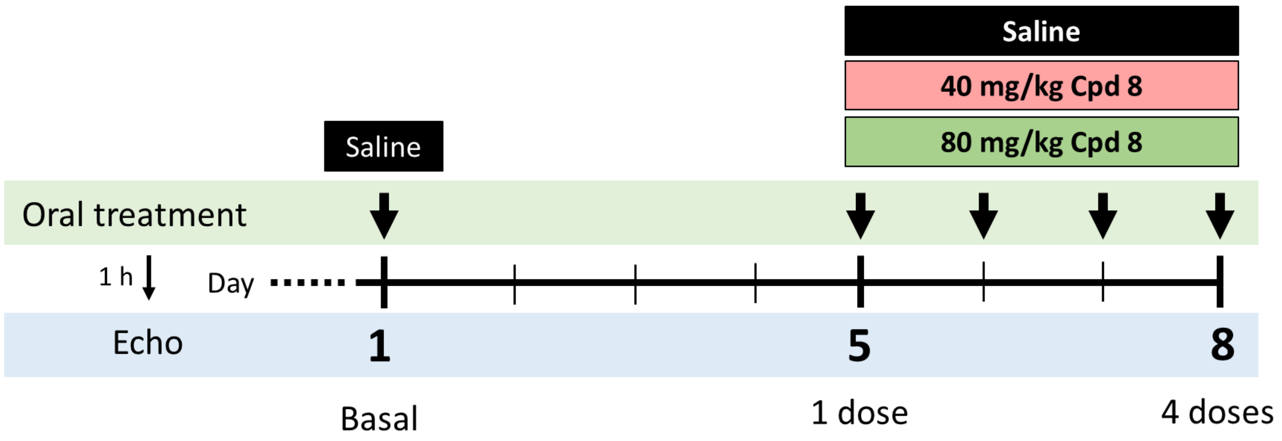

Figure S2. Protocol outline for oral treatment of STZ rats with compound 8 at 40 mg/kg or 80 mg/kg vs control group (saline). At day 1 rats were randomly assigned to either control and drug-treated groups; all the animals received saline by oral gavage and they were subjected to basal echocardiography. From day 5 to day 8, each group of rats was orally treated once daily with saline or compound 8 (40 mg/kg or 80 mg/kg); all animals were subjected to echocardiography at day 5 (1 dose) and day 8 (4 doses). Echo measurements were performed 1h following treatment under ketamine/pentobarbital anesthesia (60-37.5 mg/kg, i.p.) to permit the recovery of the animals after each experimental echocardiographic session.

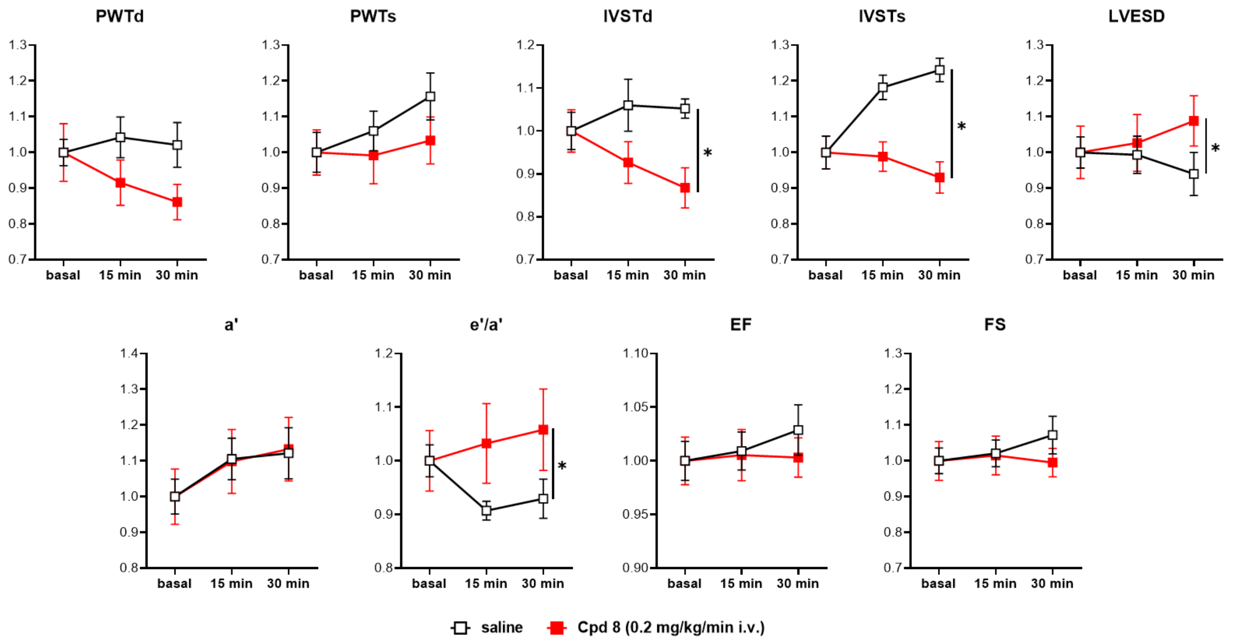

Figure S3. ***In vivo*** **effects during i.v. infusion in STZ rats.** Compound 8 was i.v. infused at 0.2 mg/kg/min under urethane anesthesia, in rats 8 weeks after STZ treatment. Echocardiographic parameters were measured under basal condition, and at 15 and 30 minutes during drug infusion. Data are mean ± SEM. Saline group N=8, compound 8 group N=11; *p<0.05 vs saline group for the interaction factor in RM two-way ANOVA. PWTd, PWTs: posterior wall thickness in diastole or systole, IVSTd, IVSTs: interventricular septum thickness in diastole or systole, LVESD: LV end-systolic diameter, a', e': late and early diastolic mitral annulus velocity; EF: ejection fraction; FS: fractional shortening. See Figure 5 of the main text for the other echo parameters.

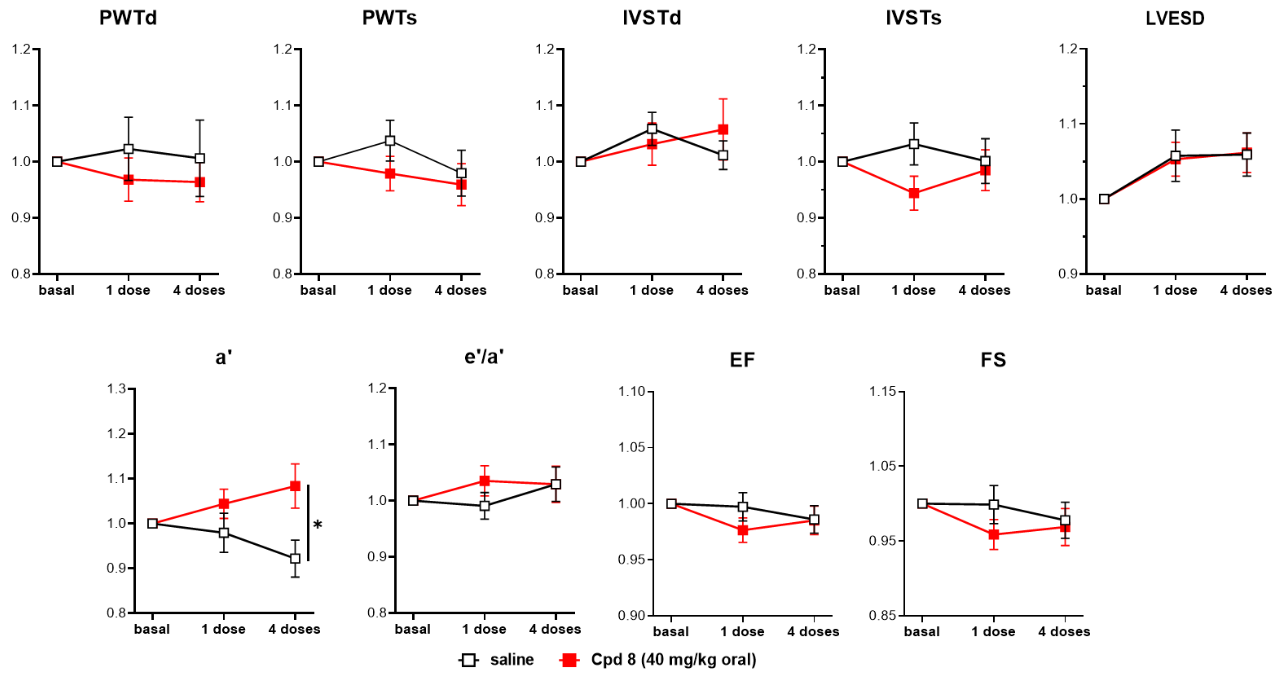

Figure S4. *In vivo* effects following oral treatment in STZ rats (40mg/kg compound 8). Rats were treated with 1 or 4 oral daily doses of compound 8 (40 mg/kg) or saline, accordingly to the protocol shown in Figure S2. Echocardiographic parameters were measured in each group 60 min post treatment under ketamine/pentobarbital anesthesia; each measurement was normalized to its basal value to highlight changes between experimental groups. Data are mean ± SEM; saline N=21, 40 mg/kg compound 8 N=22. *$p<0.05$ vs saline group for the interaction factor in RM two-way ANOVA. PWTd, PWTs: posterior wall thickness in diastole or systole, IVSTd, IVSTs: interventricular septum thickness in diastole or systole, LVESD: LV end-systolic diameter, a', e': late and early diastolic mitral annulus velocity; EF: ejection fraction; FS: fractional shortening. See Figure 6 of the main text for the other echo parameters.

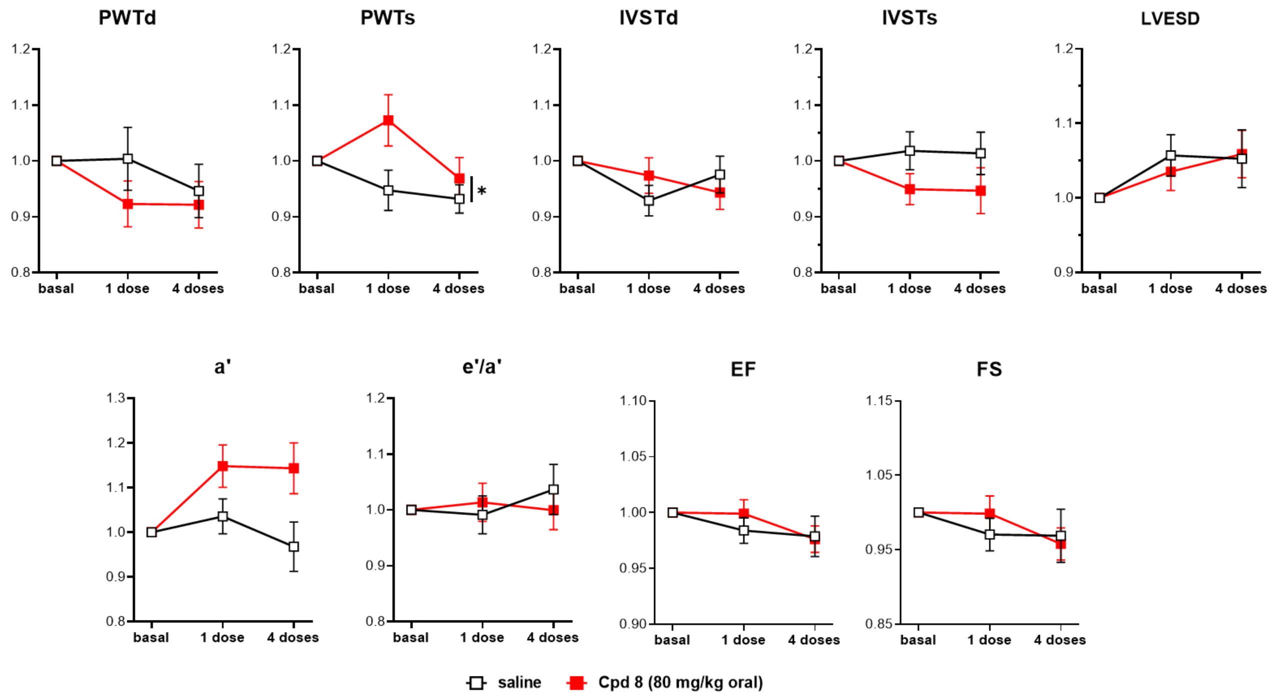

Figure S5. *In vivo* effects following oral treatment in STZ rats (80mg/kg compound 8). Rats were treated with 1 or 4 oral daily doses of compound 8 (80 mg/kg) or saline, accordingly to the protocol shown in Figure S2. Echocardiographic parameters were measured in each group 60 min post treatment under ketamine/pentobarbital anesthesia; each measurement was normalized to its basal value to highlight changes between experimental groups. Data are mean ± SEM; saline N=19, 80 mg/kg compound 8 N=21. *$p<0.05$ vs saline group for the interaction factor in RM two-way ANOVA. PWTd, PWTs: posterior wall thickness in diastole or systole, IVSTd, IVSTs: interventricular septum thickness in diastole or systole, LVESD: LV end-systolic diameter, a’, e’: late and early diastolic mitral annulus velocity; EF: ejection fraction; FS: fractional shortening. See Figure 7 of the main text for the other echo parameters.

Table S1. Effect of compound 8 (10 µM) on a panel of molecular targets (Eurofins, Taiwan). Data are reported as Δ% effect (inhibition or activation).

Cat #	Assay name	Batch	Species	Cpd 8 effect (Δ%)
107480	ATPase, Ca^{2+}, skeletal muscle	438642	pig	-1
118040	CYP450, 19	438644	human	0
124010	HMG-CoA Reductase	438610	human	-4
140010	Monoamine Oxidase MAO-A	438645	human	1
140120	Monoamine Oxidase MAO-B	438647	human	-2
143000	Nitric Oxide Synthase, Endothelial (eNOS)	438568	bovine	2
107300	Peptidase, Angiotensin Converting Enzyme	438641	rabbit	7
164610	Peptidase, Renin	438648	human	7
152000	Phosphodiesterase PDE3	438611	human	-25
171601	Protein Tyrosine Kinase, ABL1	438612	human	13
176810	Protein Tyrosine Kinase, Src	438613	human	2
200510	Adenosine A1	438614	human	-1
200610	Adenosine A2A	438614	human	-1
203100	Adrenergic α1A	438615	rat	5
203200	Adrenergic α1B	438615	rat	6
203630	Adrenergic α2A	438616	human	-2
204010	Adrenergic β1	438652	human	2
204110	Adrenergic β2	438571	human	-6
204600	Aldosterone	438617	rat	-3
206000	Androgen (Testosterone)	438618	human	6
210030	Angiotensin AT1	438653	human	1
210120	Angiotensin AT2	438653	human	-6
214600	Calcium Channel L-type, Dihydropyridine	438620	rat	-20
219500	Dopamine D1	438660	human	13
219700	Dopamine D2s	439024	human	-4
219800	Dopamine D3	438660	human	0
226010	Estrogen ERα	438622	human	-3

226050	Estrogen ERβ	438622	hum	-6
226600	$GABA_A$, Flunitrazepam, Central	438624	rat	1
226500	$GABA_A$, Muscimol, Central	438623	rat	2
232030	Glucocorticoid	438626	human	-9
233000	Glutamate, NMDA, Phencyclidine	438627	rat	-7
239610	Histamine H1	438628	human	12
241000	Imidazoline I2, Central	438629	rat	1
243000	Insulin	438654	rat	4
252710	Muscarinic M2	438621	human	-20
252810	Muscarinic M3	438661	human	-6
253010	Muscarinic M5	438661	human	0
258730	Nicotinic Acetylcholine α3β4	438656	human	-3
260410	Opiate μ (OP3, MOP)	438616	human	11
264500	Phorbol Ester	438624	mouse	-7
265600	Potassium Channel (K_{ATP})	438632	hamster	-11
265900	Potassium Channel hERG	438633	human	0
299005	Progesterone PR-B	438638	human	1
270300	Ryanodine RyR3	438634	rat	-10
271010	Serotonin (5-Hydroxytryptamine) 5-HT1, non-selective	438668	rat	12
299007	Sigma σ2	438662	human	4
278110	Sigma σ1	438636	human	2
279510	Sodium Channel, Site 2	438637	rat	-5
204410	Transporter, Norepinephrine (NET)	438597	human	-4

Table S2. Echocardiographic and tissue Doppler parameters in STZ rats at basal, after i.v. administrations of saline or compound 8 at 0.2 mg/kg/min. Raw echo parameters measured after 15 and 30 min from treatment start under urethane anesthesia. Data are mean ± SEM, N=number of rats. Statistical analysis is reported in Figures 5 and S3.

		Saline			Cpd 8 (0.2 mg/kg/min)		
		Basal	15 min	30 min	Basal	15 min	30 min
Morphometric parameters	IVSTd, mm	1,68±0,07	1,78±0,10	1,76±0,04	1,85±0,09	1,72±0,09	1,61±0,09
	PWTd, mm	1,19±0,04	1,24±0,07	1,21±0,07	1,18±0,10	1,08±0,07	1,02±0,06
	LVEDD, mm	7,53±0,09	7,66±0,13	7,68±0,14	6,95±0,11	7,22±0,13	7,47±0,16
	IVSTs, mm	2,26±0,09	2,44±0,07	2,54±0,07	2,35±0,11	2,32±0,10	2,18±0,10
	PWTs, mm	2,08±0,12	2,20±0,12	2,40±0,14	2,17±0,14	2,15±0,17	2,25±0,14
	LVESD, mm	3,74±0,16	3,71±0,20	3,51±0,23	3,10±0,23	3,18±0,25	3,37±0,22
Systolic function	FS, %	50,63±1,82	51,70±1,89	54,29±2,65	55,45±3,01	56,30±3,00	55,19±2,21
	s', mm/s	22,24±0,40	22,76±0,66	22,88±0,69	21,83±1,09	24,47±0,74	22,68±0,96
	EF, %	85,96±1,56	86,75±1,52	88,43±2,02	88,68±1,97	89,16±2,12	88,96±1,61
Diastolic function	E, mm/s	0,78±0,05	0,82±0,04	0,86±0,05	0,84±0,05	0,90±0,04	0,87±0,03
	A, mm/s	0,56±0,05	0,64±0,05	0,66±0,05	0,56±0,04	0,66±0,05	0,68±0,04
	E/A	1,41±0,08	1,32±0,07	1,32±0,07	1,59±0,12	1,42±0,08	1,33±0,08
	DT, ms	60,25±3,17	57,63±2,38	58,88±2,23	58,46±3,37	49,73±3,79	47,46±3,07
	DT/E, s^2/mm	79,07±5,25	71,18±4,46	70,29±4,98	71,90±5,83	56,52±4,96	54,80±3,87
	E/DT, mm/s^2	13,02±0,82	14,46±0,94	14,86±1,30	15,51±2,10	19,79±2,49	19,38±1,63
	e', mm/s	21,14±0,78	21,27±0,95	21,84±0,82	20,71±0,57	23,1±0,59	24,48±0,63
	a', mm/s	25,48±1,25	28,16±1,48	28,56±1,82	23,54±1,82	25,86±2,10	26,66±2,09
	e'/a'	0,84±0,02	0,75±0,01	0,78±0,03	0,92±0,05	0,95±0,07	0,97±0,07
	E/e'	36,66±1,56	38,94±1,67	39,36±1,58	40,66±1,65	39,08±1,52	35,82±1,18
Overall cardiac function	HR, bpm	230,5±7,4	231±7,4	235±11,1	223,1±18,3	221,9±15,1	224,6±15,0
	SV, ml	0,82±0,03	0,87±0,03	0,89±0,04	0,68±0,03	0,76±0,04	0,83±0,04
	CO	189±9.2	200.5±9,4	208±12,8	149,4±10,1	165,5±9,3	181,8±7,2
	N	8	8	8	11	11	11

Table S3. Echocardiographic and tissue Doppler parameters in STZ rats after 1 or 4 oral daily administrations of saline or compound 8 at 40 mg/kg. Raw echo parameters measured after 60 min from treatment start under ketamine/pentobarbital anesthesia. Data are mean ± SEM, N=number of rats. Statistical analysis is reported in Figures 6 and S4.

		Saline			Cpd 8 (40 mg/kg)		
		Basal	1 dose	4 doses	Basal	1 dose	4 doses
Morphometric parameters	IVSTd, mm	1,61±0,05	1,69±0,05	1,63±0,04	1,57±0,05	1,6±0,04	1,63±0,04
	PWTd, mm	1,03±0,04	1,03±0,04	1,02±0,05	1,03±0,03	0,99±0,04	0,98±0,03
	LVEDD, mm	7,85±0,14	8,13±0,13	8,09±0,12	8,30±0,11	8,38±0,09	8,42±0,11
	IVSTs, mm	2,10±0,07	2,14±0,06	2,10±0,07	2,19±0,08	2,04±0,06	2,13±0,08
	PWTs, mm	1,77±0,06	1,81±0,06	1,73±0,07	1,79±0,05	1,74±0,06	1,70±0,07
	LVESD, mm	4,08±0,13	4,26±0,12	4,28±0,13	4,44±0,10	4,65±0,09	4,67±0,10
Systolic function	FS, %	48,15±1,04	47,77±1,02	46,95±1,21	46,57±0,93	44,45±0,89	44,75±0,79
	s', mm/s	21,37±0,54	20,76±0,55	20,70±0,57	20,72±0,39	21,2±0,44	21,57±0,42
	EF, %	83,85±0,89	83,47±0,83	82,64±1,05	82,39±0,85	80,36±0,89	80,95±0,66
Diastolic function	E, mm/s	0,95±0,03	0,91±0,02	0,86±0,03	0,85±0,02	0,87±0,03	0,92±0,03
	A, mm/s	0,78±0,04	0,72±0,03	0,68±0,03	0,68±0,03	0,71±0,03	0,76±0,03
	E/A	1,27±0,05	1,30±0,04	1,29±0,04	1,28±0,04	1,24±0,03	1,23±0,03
	DT, ms	53,33±2,17	59,48±1,87	56,95±1,78	57,41±2,50	55,09±2,06	56,14±2,3
	DT/E, s^2/mm	58,24±3,89	66,59±2,84	67,37±2,87	69,03±3,84	65,0±3,58	63,12±3,66
	E/DT, mm/s^2	18,88±1,35	15,66±0,76	15,42±0,73	15,75±1,14	16,62±1,13	17,55±1,61
	e', mm/s	23,29±0,61	21,97±0,49	21,52±0,37	21,49±0,50	22,8±0,52	23,28±0,57
	a', mm/s	28,54±1,06	27,28±0,93	25,88±1,02	26,82±0,96	27,8±1,08	28,69±1,23
	e'/a'	0,83±0,02	0,81±0,02	0,85±0,03	0,81±0,02	0,84±0,03	0,83±0,03
	E/e'	40,97±1,05	41,49±1,08	40,07±1,13	39,75±0,88	38,39±0,98	39,51±0,79
Overall cardiac function	HR, bpm	249,5±12,0	222,2±8,4	220,8±8,3	221,4±9,4	232,4±8,8	240,6±9,2
	SV, ml	0,90±0,04	0,99±0,04	0,96±0,04	1,03±0,04	1,02±0,03	1,05±0,04
	CO	221,8±11,8	219,5±11,1	217,7±13,7	228±12,3	240,0±13,2	252,8±12,1
	N	21	21	20	22	22	21

Table S4. Echocardiographic and tissue Doppler parameters in STZ rats after 1 or 4 oral daily administrations of saline or compound 8 at 80 mg/kg. Raw echo parameters measured after 60 min from treatment start under ketamine/pentobarbital anesthesia. Data are mean ± SEM, N=number of rats. Statistical analysis is reported in Figures 7 and S5.

		Saline			Cpd 8 (80 mg/kg)		
		Basal	1 dose	4 doses	Basal	1 dose	4 doses
Morphometric parameters	IVSTd, mm	1,72±0,05	1,58±0,04	1,66±0,05	1,69±0,03	1,63±0,04	1,58±0,054
	PWTd, mm	1,06±0,05	1,04±0,05	0,97±0,04	1,11±0,03	1,01±0,04	1,02±0,05
	LVEDD, mm	8,12±0,12	8,23±0,13	8,15±0,13	8,38±0,12	8,52±0,10	8,43±0,15
	IVSTs, mm	2,18±0,07	2,19±0,06	2,18±0,06	2,27±0,07	2,14±0,07	2,11±0,07
	PWTs, mm	2,13±0,05	1,99±0,06	1,97±0,06	2,00±0,08	2,10±0,07	1,93±0,08
	LVESD, mm	4,27±0,11	4,47±0,10	4,45±0,15	4,48±0,11	4,60±0,09	4,67±0,15
Systolic function	FS, %	47,42±1,03	45,74±0,79	45,56±1,33	46,56±1,04	46,10±0,69	44,88±1,11
	s', mm/s	21,45±0,46	21,94±0,50	20,69±0,53	21,24±0,45	22,20±0,3	22,47±0,55
	EF, %	83,19±0,88	81,73±0,73	81,26±1,22	82,21±0,96	82,04±0,67	80,71±1,04
Diastolic function	E, mm/s	0,91±0,03	0,88±0,03	0,85±0,03	0,86±0,02	0,95±0,02	0,93±0,03
	A, mm/s	0,68±0,04	0,69±0,03	0,64±0,03	0,68±0,02	0,77±0,03	0,76±0,04
	E/A	1,38±0,05	1,30±0,03	1,35±0,04	1,29±0,02	1,26±0,03	1,27±0,06
	DT, ms	58,68±2,97	56,42±2,89	57,95±2,32	60,62±2,11	55,86±2,63	49,11±2,58
	DT/E, s^2/mm	66,78±4,63	65,00±3,65	69,65±3,44	70,52±2,26	60,21±3,52	54,60±3,70
	E/DT, mm/s^2	16,80±1,58	16,61±1,27	15,09±0,88	14,57±0,64	18,57±1,76	20,35±1,91
	e', mm/s	23,85±0,75	23,82±0,48	22,71±0,68	22,06±0,47	25,04±0,59	24,63±0,92
	a', mm/s	27,74±1,27	28,20±1,04	25,96±1,15	26,50±0,92	29,86±0,86	30,18±1,46
	e'/a'	0,87±0,02	0,86±0,03	0,90±0,03	0,84±0,02	0,85±0,02	0,83±0,02
	E/e'	38,38±0,92	37,23±1,17	37,80±1,53	39,23±0,66	38,09±0,82	37,99±1,07
Overall cardiac function	HR, bpm	232,2±12,8	230,1±11,5	236,6±10,7	229,1±9,8	248,1±10,1	262,3±11,6
	SV, ml	0,98±0,04	1,00±0,04	0,97±0,04	1,06±0,04	1,10±0,04	1,05±0,04
	CO	224,2±12,0	226,9±12,0	228,6±13,7	241±13	271±11	274±15
	N	19	19	19	21	21	18

References

Alemanni, M., Rocchetti, M., Re, D., and Zaza, A. (2011). Role and mechanism of subcellular Ca2+ distribution in the action of two inotropic agents with different toxicity. J. Mol. Cell. Cardiol. *50*: 910–918.

Rocchetti, M., Besana, A., Mostacciuolo, G., Ferrari, P., Micheletti, R., and Zaza, A. (2003). Diverse toxicity associated with cardiac Na+/K+ pump inhibition: Evaluation of electrophysiological mechanisms. J. Pharmacol. Exp. Ther. *305*: 765–771.

Rocchetti, M., Besana, A., Mostacciuolo, G., Micheletti, R., Ferrari, P., Sarkozi, S., et al. (2005). Modulation of sarcoplasmic reticulum function by Na+/K + pump inhibitors with different toxicity: Digoxin and PST2744 [(E,Z)-3-((2-aminoethoxy)imino)androstane-6,17-dione hydrochloride]. J. Pharmacol. Exp. Ther. *313*: 207–215.

Torre, E., Arici, M., Lodrini, A.M., Ferrandi, M., Barassi, P., Hsu, S.-C., et al. (2022). SERCA2a stimulation by istaroxime improves intracellular Ca2+ handling and diastolic dysfunction in a model of diabetic cardiomyopathy. Cardiovasc. Res. *118*: 1020–1032.
